# The Use of Samsung Health and ECG M-Trace Base II Applications for the Assessment of Exercise Tolerance in the Secondary Prevention in Patients after Ischemic Stroke

**DOI:** 10.3390/ijerph18115753

**Published:** 2021-05-27

**Authors:** Mateusz Lucki, Ewa Chlebuś, Agnieszka Wareńczak, Przemysław Lisiński

**Affiliations:** 1 Department of Cardiology, Hospital Center of the Jelenia Góra Valley, Poland Ogińskiego Str. No. 6, 58-506 Jelenia Góra, Poland; 2 Department of Rehabilitation and Physiotherapy, University of Medical Sciences, 28 Czerwca 1956 Str. No. 135/147, 60-545 Poznań, Poland; ewachlebus@ump.edu.pl (E.C.); agnieszka.warenczak@gmail.com (A.W.); plisinski@vp.pl (P.L.)

**Keywords:** applications, Samsung Health, ECG M-Trace Base II, exercise tolerance, secondary prevention, ischemic stroke

## Abstract

**Background and objectives**: The aim of the study was to use the mobile application Samsung Health for the assessment of parameters of exercise tolerance and the ECG (electrocardiogram) M-Trace Base II for the assessment of cardiological parameters. **Materials and Methods**: The measurements were conducted during rest and after performing SMWT (Six Minute Walk Test) and SCT (Stair Climb Test) in 26 patients after ischemic stroke (IS) and 26 healthy individuals. **Results**: In the SMWT, the post-stroke group (SG) walked a shorter distance (*p* < 0.001), achieving lower mean gait velocity (*p* < 0.001) and lower maximum gait velocity (*p* = 0.002). In the SCT, SG achieved a lower mean gait velocity (*p* < 0.001) and lower maximum gait velocity (*p* < 0.001) when compared to the control group (CG). In SG, myocardial ischemia in ECG was noted in four patients after SMWT and in three patients following SCT. Both in SG and in CG the increase in SBP (systolic blood pressure) value measured after SMWT and SCT compared to at rest (*p* < 0.001) was observed. In SG, in the compared ratios rest to SMWT and SCT as well as SMWT to SCT, there was an increase in HR (heart rate) (*p* < 0.001). **Conclusions**: ECG M-Trace Base II and Samsung Health are mobile applications that can assess cardiological parameters and exercise tolerance parameters in patients after IS, so they can be used to plan the intensity of exercise in rehabilitation programs.

## 1. Introduction

Stroke is a significant social problem, because it remains one of the main causes of morbidity and long-term disability and the second most frequent cause of death [1,2,3]. Currently, a significant increase in stroke prevalence is observed and approximately 31% of stroke events occur in people under 65 years of age [2,3]. Patients with stroke in their medical history are a group at high risk of reoccurrence of subsequent cardiovascular disease (CVD) events [4]. Widely recognized, modifiable CVD risk factors in the secondary prevention include concomitant diseases, such as: arterial hypertension, diabetes, dyslipidemia, obesity, atrial fibrillation, internal carotid artery stenosis, depression, insomnia, and unhealthy lifestyle such as: cigarette smoking, alcohol overuse, limited physical activity [5,6,7,8,9,10,11,12,13,14]. The above-mentioned limitation of physical activity is responsible for the occurrence of approximately 8% of strokes [15]. Literature data confirm that regular physical exercise favorably modifies the risk factors for vascular diseases, including stroke, by decreasing blood pressure and body weight, and it has a beneficial effect on glucose tolerance [16].

Patients after stroke are often burdened with concomitant cardiac diseases [17]. It is worth noting that rehabilitation of patients after stroke is usually focused on reducing the dysfunctions caused by neurological deficits, but it does not include potential consequences associated with training load [18]. Due to the existing neurological deficits or contraindications for it in post-stroke patients, it is difficult to perform exercise stress test on a treadmill or a cycloergonometer [19]. Therefore, it is very important to search for other methods of assessment of exercise tolerance in post-stroke patients.

The progress of information technologies, including the popularity of applications monitoring, among others, heart rate, the number of calories burned depending on the type of physical activity or enabling ECG (electrocardiogram) recording allows to control the primary parameters of health status [20]. Studies have shown that using mobile applications is becoming effective in treatment monitoring [21,22]. In the available literature, no mobile applications have been used in clinical practice to assess the tolerance of efforts and cardiological parameters during Six-Minute Walk Test (SMWT) and Stair Climb Test (SCT).

The purpose of these examinations was to assess:

1. Exercise tolerance parameters in patients after ischemic stroke (IS) during SMTW i SCT with the use of mobile application Samsung Health.
2. Cardiological parameters in patients after ischemic stroke (IS) during SMTW i SCT with the use of mobile application ECG M-Trace Base II.
3. Risk factors for CVD events recurrence in patients after IS in secondary prevention.

Research hypotheses:

1. We assume that patients who have undergone IS have gotten worse results of cardiological parameters and exercise tolerance parameters during SMTW and SCT compared to patients without previous IS.
2. We assume that the M-Trace Base II and the Samsung Health are popular mobile applications that can be used to assess cardiological parameters and exercise tolerance parameters during SMWT and SCT in patients after IS.
3. We assume that there are significant numbers of risk factors for recurrent CVD in patients after IS.

## 2. Materials and Methods

### 2.1. Study Design

**Figure 1:**
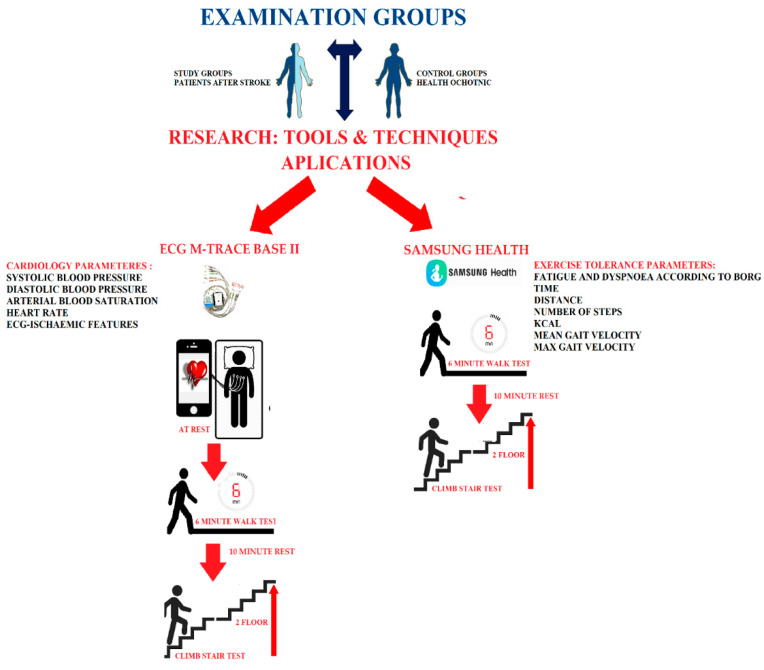
Study design.

In the design of this study, two research groups were created consisting of a post-stroke group and a control group without a stroke. All participants were tested in the same way. Each group first performed a trial SMWT and SCT. The next day, SMWT and SCT were performed again, the results of which are presented in our article. In the study we used the mobile ECG M-Trace Base II and Samsung Health applications. With the Samsung Health application, exercise tolerance parameters such as time of test performance, the walked distance and steps made, as well as the mean gait velocity and maximum gait velocity and burned calories were assessed. Additionally, after the tests performed, the level of dyspnea and fatigue was assessed according to the modified Borg scale. The evaluation of these parameters was performed after SMTW and SCT. Using the ECG M-Trace Base II application, cardiological parameters such as ECG and HR (heart rate) were assessed. In the assessment of cardiological parameters, the blood pressure monitor was additionally used to measure the systolic (SBP) and diastolic blood pressure (DBP) as well as the pulse oximeter to measure the saturation of arterial blood with oxygen. The evaluation of these parameters was performed at rest, after SMTW and after SCT. The design of this study is shown in Figure 1.

### 2.2. Materials

The study group (post-stroke) consisted of 26 patients (mean age 54.9 ± 7.1) in whom ischemic stroke (IS) occurred within 14 days before the admission to the Clinical Department and who were hospitalized at the Department of Neurological Rehabilitation of the Clinical Department of Rehabilitation in the Wiktor Dega Orthopaedic and Rehabilitation Clinical Hospital in Poznań from November 2020 to January 2021. The examination was performed within 3 days of being admitted to the Department.

Inclusion criteria to the post-stroke group were as follows: (1) occurrence of hemiparesis after the first episode of IS (2) IS confirmed in the diagnostic imaging records (3) score >3 on Manual Muscle Test (MMT) which means that patient is capable to do active movement against gravity in the paretic limbs (4) score ≥21 in Barthel’s scale which means that patient does not need or needs only partial help in daily leaving activity (5) score <16 in the National Institutes of Health Stroke Scale (NIHSS) which means that patient has undergone at most moderate IS (6) available complete medical records concerning the assessed risk factors of recurrent CVD event.

Exclusion criteria from the post-stroke group were as follows: (1) hemiparesis or tetraparesis following many episodes of IS, (2) lack of diagnostic imaging scans confirming the occurrence of IS (3) hospital stay at the Department of Neurological Rehabilitation for more than 14 days from the occurrence of IS (4) incomplete medical records concerning the assessed risk factors of recurrent CVD event (5) score <3 on MMT which means that patient is not capable to do active movement against gravity in the paretic limbs (6) score <21 in Barthel’s scale which means that patient needs total help in daily leaving activity (7) score ≥16 in the NIHSS which means that patient has undergone severe IS (8) occurrence of factors limiting walking ability, such as diagnosed pulmonary diseases, unstable angina pectoralis, severe valvular heart diseases, cardiomyopathy, musculoskeletal or autoimmune diseases.

In order to obtain reference values for the results of studies carried out in patients after stroke, the same tests were performed in a control group consisting of 26 volunteers out of hospital employees with a similar distribution of sex and age (mean age 55.6 ± 6.1). Information about recruitment appeared on the hospital’s website; the health condition of the volunteers was determined based on medical examination. They have not had a stroke in the past, were in good general physical health and did not show signs of unstable angina, severe heart valvular defects, cardiomyopathy, neurological diseases, musculoskeletal disorders, autoimmune or respiratory diseases in clinical examination.

On the basis of available medical records we assessed occurrence of the risk factors for reoccurrence of a CVD event, such as: arterial hypertension (>140/90 mmHg), atrial fibrillation, diabetes mellitus (HbA1C > 7 mg%), dyslipidemia (LDL > 55 mg/dL), internal carotid artery stenosis (>50%), depression (Beck Depression Inventory score > 19), insomnia(sleep time <6 or >9 h), BMI (body mass index) value > 25, nicotinism and alcoholism (alcohol intake per day >10 g) [5,6,7,8,9,10,11,12,13,14]. A detailed presentation of groups is provided in Table 1.

### 2.3. Variables and Instruments

Samsung smartphones were the first to have the Health application enabling tracking the measurements associated with physical activity. It was the first free application available for all the Android users. Since 2 October 2017, this application has been available for iPhones with iOS 9.0 (Apple Inc., Cupertino, CA, USA) operating system [23]. Samsung Health is an accurate tool that serves to, among others, count the steps in various age groups and regardless the gait pace, even during slow walking. As it has been proved in the studies by Johnson et al. [24], Samsung Health is the most accurate application in detecting steps and energy expenditure during a self-selected walking pace. This application provides information on the walked distance, mean and maximum gait velocity, the number of steps, and the number of calories burned. Moreover, thanks to the additional equipment, it enables monitoring heart rate and arterial blood saturation. In addition, the application contains, among others, the modules for monitoring body mass, sleep, water use, and caffeine consumption.

**Figure 2:**
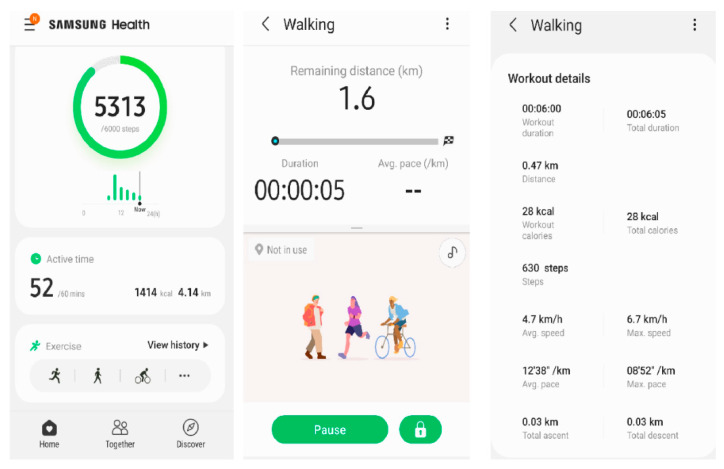
Samsung Health application interface.

Measurements were performed on Samsung Galaxy S9 smartphone. The Samsung Health application version 6.15.1.003 (Samsun Electronics Co., Suwon, South Korea) was used. When performing the SMWT and SCT, gait measurement with pedometer function was used. The following parameters were monitored: activity time (minutes), walked distance (meters), number of steps, mean gait velocity (km/h) and maximum gait velocity (km/h), and number of calories burned (kcal). During the tests, the study participant held smartphone in right hand. Additionally, after each test, the level of dyspnoea and fatigue was assessed according to the modified Borg scale (0—nothing at all, from 1 to 2—weak, from 3 to 4—moderate, from 5 to 6—strong, from 7 to 9—very strong, 10—extremely strong). Figure 2 presents application report interface.

**Figure 3:**
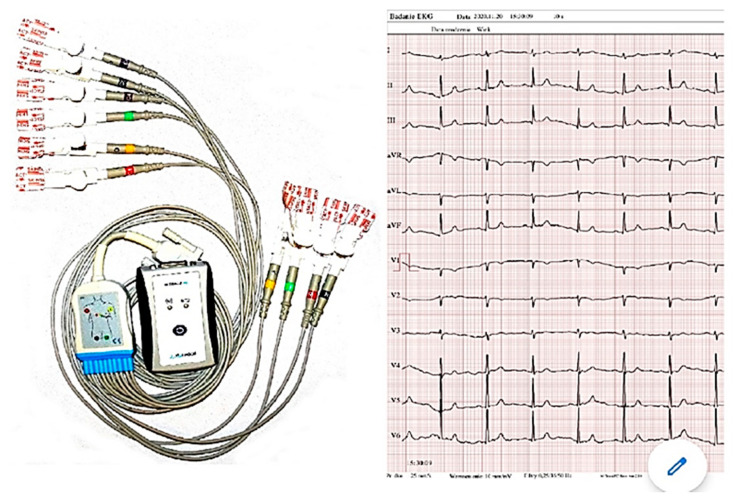
ECG M- Trace Base II.

ECG M-Trace Base II application is a software designed to record and assess the ECG recordings in mobile phones with Android operating system. In order to record the ECG results, the application requires the M-Trace recorder which is sold together with the software. The M-Trace Base II (c) recorder was developed according to the requirements of the 93/42/EEC directive and PN-EN 62304 standard, which determines the guidelines on designing and development of medical systems. The M-Trace recorder has compact design, small size, and internal power supply. It communicates with smartphone via wireless Bluetooth standard connection. In connection with the application, the ECG M-Trace Base II recorder (Figure 3) is a device intended for full ECG recording in 12 standard leads [25]. After connecting the electrodes, the device sends a signal to the smartphone application, where it is possible to read and interpret the ECG record.

**Figure 4:**
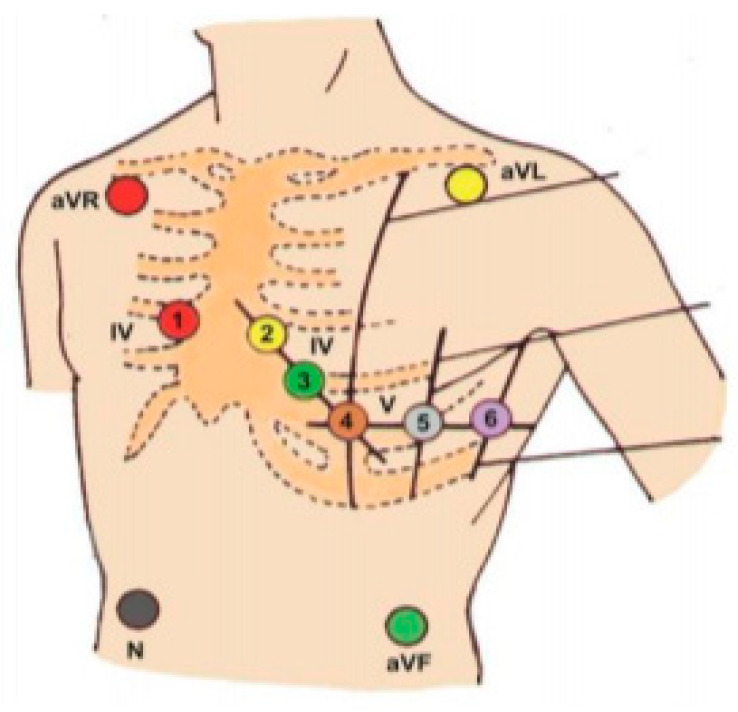
Electrode placement scheme.

In order to perform the correct ECG recording, the electrodes of the device should be placed according to the diagram presented in Figure 4.

Bipolar Einthoven limb leads (4 electrodes):

- red electrode—right hand.
- yellow electrode—left hand.
- green electrode—left shin.
- black electrode—right shin (the so-called reference point; Earth).

Augmented Goldberger unipolar limb leads:

- aVR lead—from the “right hand” electrode.
- aVL lead—from the “left hand” electrode.
- aVF lead—from the “left shin” electrode.

Unipolar Wilson precordial leads (6 electrodes):

- V1—electrode on the fourth right intercostal space at the sternal edge.
- V2—electrode on the fourth left intercostal space at the sternal edge.
- V3—halfway between V2 and V4 electrodes.
- V4—electrode on the fifth left intercostal space at the left midclavicular line.
- V5—electrode on the fifth left intercostal space at left anterior axillary line.
- V6—electrode on the fifth left intercostal space at the left midaxillary line.

ECG recordings were performed with the use of ECG M-Trace Base II device with the assessment of heart rate (per minute) and ischemic features at rest, after completion of SMWT and SCT. In addition, cardiological parameters such as SBP and DBP (mmHg), HR (per minute), and arterial oxygen saturation (%) were assessed. Conditions of performing SMWT were in accordance with the protocol of the American Thoracic Society [26]. The test was conducted at the doctor’s presence for each of the study participants individually, on an empty corridor with marked start and turn, of length 30 m. During the test, the patient walked at his own pace for 6 min. The patient could use orthopedic aids (walking frame, crutches, walking stick) and have breaks due to exhaustion, limiting dyspnoea, muscle weakness, or chest pain. Oral motivation to continue the exercise was used during the test.

The SCT was conducted at the doctor’s presence, on an empty staircase for each of the study participants individually. Each floor had 23 stairs of height 0.18 m. The test consisted in climbing a total of 46 stairs in order to reach the 2nd floor. A total walking distance was 50 m. It could use orthopedic aids (walking frame, crutches, walking stick) and have breaks due to exhaustion, limiting dyspnea, muscle weakness, or chest pain. Oral motivation to continue the exercise was used during the test.

Performing SMTW and SCT was preceded by a 10-min rest in a sitting position.

### 2.4. Statistical Analysis

Data were analyzed with Statistica version 13.1. (StatSoft Co., Cracow, Poland) Descriptive statistics were reported as means, standard deviations (SD), median, minimum, and maximum. The categorical variables were presented as counts and frequencies. The Shapiro–Wilk test was used to assess the normality of distributions in the test score. Nonparametric analyses were used when the data did not meet the assumptions for parametric analysis. To assess the significance of the differences between the results of the study (stroke group) and control groups, the parametric Student’s t-test, Welch test (with lack of variance homogeneity), or nonparametric Mann–Whitney test were used. Nonparametric Friedman test was used to determine whether there were any differences between the three measurements (during rest, after SMWT, and after SCT). A post-hoc analysis was used in the cases when there were statistically significant differences in the measures. The chi-square test was used to compare difference between groups for categorical variables. *p*-values of less than 0.05 were considered statistically significant.

### 2.5. Research Ethics

The study was conducted in accordance with ethical principles for biomedical rese-arch as stated in the Declaration of Helsinki. Each test person was informed about the purpose and methodology of the research and gave informed consent to participate. The study was prospective and approved by the Ethics Committee at the Karol Marcinkowski Memorial Medical University in Poznań (Approval No. 175/21 of 11 March 2021). The study was conducted in accordance with ethical principles for biomedical research as stated in the Declaration of Helsinki. The study was registered in the Clinical Trial Registry: NCT04821518 https://clinicaltrials.gov/ct2/show/NCT04821518 (accessed on 29 March 2021).

## 3. Results

### 3.1. Risk Factors for the Reoccurrence of a Cardiovascular Disease Event

In the post-stroke group, a significantly higher BMI value (*p* < 0.001), concomitant nicotinism (*p* = 0.03), arterial hypertension (*p* < 0.001), diabetes (*p* = 0.03), dyslipidemia (*p* < 0.001), ICA stenosis (*p* = 0.018), depression (*p* = 0.030), and insomnia (*p* = 0,002) were observed compared to the control group. Additionally, in the post-stroke group the patients took β- blockers more often (*p* < 0.001). A detailed distribution of the occurrence of risk factors for reoccurrence of a CVD event is presented in Table 1.

### 3.2. Exercise Tolerance Parameters Measured with Samsung Health Application

Table 2 shows detailed results of exercise tolerance parameters depending on the type of exercise test. Both during SMWT and SCT, in the post-stroke group of patients, significantly greater fatigue and dyspnoea according to the modified Borg scale were observed compared to the control group (*p* < 0.001). In the post-stroke group, time of performing both SMWT (*p* = 0.044) and SCT (*p* < 0.001) was longer. In SMWT, there was a significant difference in the walked distance between the study groups (*p* < 0.001). Patients after IS walked on average 320 metres, whereas patients from the control group on average walked 550 metres, which was a result of significantly lower mean (*p* < 0.001) and maximum gait velocity (*p* = 0.002) in patients after IS. In addition, in SCT in the post-stroke group the mean (*p* < 0.001) and maximum gait velocity (*p* < 0.001) was significantly lower, whereas the number of steps when walking the same distance was significantly higher (*p* < 0.001) in comparison to the control group. The studied groups of patients differed significantly in the number of calories burned (*p* < 0.001). In SMWT a greater number of calories were burned by the control group, whereas in SCT more were burned by the post-stroke group.

### 3.3. Cardiological Parameters in the Assessment of Exercise Tolerance Partially Measured with ECG M-Trace Base Application

Table 3 presents detailed results of the assessed cardiological parameters. Analyzing the results between the post-stroke group and the control group, significant differences in arterial blood saturation were detected. In the post-stroke group lower blood saturation at rest (*p* = 0.015), after SMWT (*p* = 0.023) and SCT (*p* = 0.008) was observed as well as lower heart rate (HR) after SMWT (*p* = 0.004) and SCT (*p* < 0.001) compared to the control group. Moreover, in the post-stroke group features of ischemia in the form of significant ST-segment depressions in at least two adjacent leads, not appearing in the resting ECG, were observed in four patients after SMWT and in three patients after SCT. These patients did not report chest pain.

When analyzing the results depending on the tests performed in both the post-stroke group and the control group, a significant increase in SBP measured after SMWT and SCT was observed compared to the value measured at rest (*p* < 0.001). What is more, in the control group, the SBP value was significantly higher after SCT compared to SMWT (*p* < 0.001). This relation was not observed in the post-stroke group. A significant increase in the DBP value was noted only in the control group, when comparing between the values measured at rest and after SCT (*p* = 0.005).

In the post-stroke group, in the compared ratios rest to SMWT and SCT as well as SMWT to SCT there was a significant increase in HR (*p* < 0.001). Similar results were obtained in the control group, but a significant relation between SMWT and SCT was not observed.

**Figure 5:**
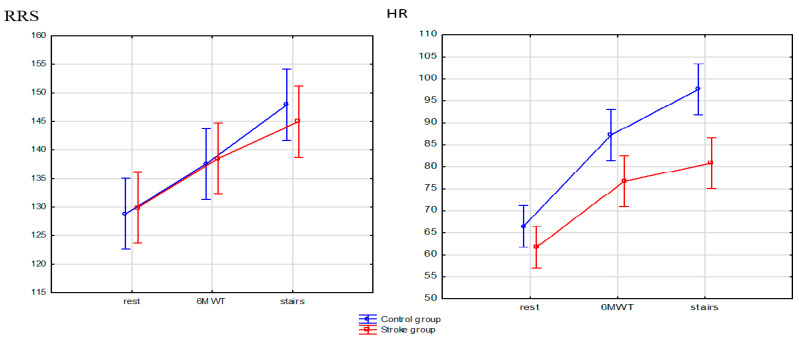
Distributions of arterial blood pressure and heart rate values.

Distributions of arterial blood pressure and heart rate values between the post-stroke group and the control group are presented in Figure 5. In the control group, higher maximum values and lower minimum SBP values compared to the post-stroke group were observed both at rest and after SMWT and SCT. The same results were obtained when assessing the HR parameter. The most pronounced difference in SBP and HR between the analyzed groups was demonstrated during the measurement after SCT.

## 4. Discussion

Using the Samsung Health application, we assessed exercise tolerance in patients after IS in SMWT and SCT. In the post-stroke group, greater fatigue and dyspnea according to Borg scale were observed both during SMWT and SCT. As it has been proved in the studies by Johnson et al. [24], Samsung Health is the most accurate application in detecting steps and energy expenditure during walking which affects the accuracy of the results obtained during SMWT and SCT. In line with the results of studies by Boujibar et al. [27], fatigue and dyspnea were greater after performing SCT; however, those authors conducted their tests in a group of patients with lung cancer. The analysis of the available subject literature revealed that SCT has never been used in the post-stroke patients so far. On the other hand, our results concerning fatigue and dyspnoea according to Borg scale during SMWT are the same as the results by Lacroix [28] et al., who examined patients after IS during convalescence at physiotherapy sessions.

Our studies showed lower gait velocity in patients after IS. Both during SMWT and SCT, lower mean and maximum gait velocity was achieved. This observation is in line with the results obtained by Jarvis et al. [29]. In addition, during SMWT there was a significant difference in the walked distance between the study groups. The post-stroke patients walked on average 320 m, whereas the control group walked on average 550 m. Similar results were shown by Bohannon et al. [30]. In our studies, a significant difference in the number of calories burned depending on the type of exercise was observed as well. Our results are in line with the studies by Polese et al. [31], who have proved that performing SMWT and SCT by patients after IS requires greater energetic costs. Moreover, in the performed tests, there was a difference in the number of steps made between the post-stroke group and the control group. During SMWT, the patients after IS made a lesser number of steps than the healthy individuals. Most likely, it was related to the shorter distance walked by these patients compared to the control group. Similar results were obtained by Fulck et al. [32], who observed a lower mean daily number of steps made by patients after IS. On the other hand, during SCT patients from the post-stroke group, when compared to the control group, made more steps when walking the same distance, which could be due to impaired motor coordination in people who underwent IS, that required making a greater number of smaller steps, as well as increased difficulty of SCT compared to SMWT [33].

With the use of ECG M-Trace Base II application, we studied the effect of SMWT and SCT on the assessment of cardiological parameters such as ECG, SBP, DBP, HR, and arterial blood saturation in patients after IS. The studied groups of patients differed significantly in the value of arterial blood saturation both at rest and after SMWT and SCT. According to Modai et al. [34], the maximum rate of oxygen consumption (VO2max) occurs after performing SCT. In our study, the lowest arterial blood saturation level was also observed after SCT. The post-stroke group and the control group did not differ significantly in HR value measured at rest. However, HR measurements after SMWT and SCT differed significantly within the groups. In the post-stroke group of patients, a smaller increase in HR was observed after performing the tests. These results could have been affected by the use of β-blockers. Taking them may decrease the heart rate by 30–35% [35]. Additionally, a smaller increase in SBP was observed with the increase in HR. The increase in SBP did not differ between the groups under the same study conditions but differed significantly within the same study group between the SBP values measured after SMWT and SCT tests compared to the values measured at rest. In the subject literature, there are not any published results of studies assessing the SBP value during SMWT and SCT. Data from the available literature have shown an increase in SBP value during and after the completion of exercise stress test [36] as well as during exercise in patients after IS [37]. In our studies, in the post-stroke group, signs of myocardial ischemia were observed in the ECG performed after SMWT and SCT, which were not present in resting ECG. Gäverth et al. [38] in the meta-analysis of monitoring of ECG recording in patients after IS during rehabilitation reported that in 10% of the analyzed studies there were cardiac arrhythmias in the ECG recording during rehabilitation exercises, which were not observed in resting ECG recording. Therefore, it seems reasonable to monitor the ECG with the use of the proposed application before enrolment in the rehabilitation program.

In our studies, in patients after stroke the coincidence of risk factors for the reoccurrence of a CVD event was observed. Our results are in line with the studies by the other authors, who proved that CVD events occur significantly more frequently depending on the occurrence of arterial hypertension [9], dyslipidemia [11], carotid atherosclerosis [8], high BMI value [14], insomnia [6], post-stroke depression [5], and nicotinism [13].

In our study, in clinical conditions, Samsung Health and ECG M-Trace Base II mobile applications were used to assess exercise tolerance and cardiological parameters, which can help adjust appropriate rehabilitation program and oversee the physical activity of patients. Smartphone applications are becoming an important part of a patient–doctor relationship in the 21st century [39]. Almost 3.8 billion people around the world have a smartphone today, which means that mobile health (m-health) provides a perspective for performing effective and cheap health services for large populations, both locally and globally [40]. Health-associated applications ensure an effective method for supporting self-treatment of, e.g., arterial hypertension [41]. Prevention of cardiovascular diseases (CVD) by the identification of people from the risk groups is a well-established, but expensive strategy, if it is based on the measurements on the basis of laboratory analyses. As demonstrated by Surka et al. [42], using mobile applications for the assessment of risk of CVD in mobile phones is burdened with a lower risk of errors in calculating the hazard ratio when compared to using the tools for CVD risk assessment in paper form. There is scientific evidence for the effectiveness of the ECG application in the assessment of risk of a cardiovascular event [43]. As it has been proved in the studies by Dolezal et al. [44], the ECG mobile application is a simple, time- and money-saving tool for the remote identification of people who potentially are at higher risk of death due to cardiovascular diseases. The m-health technologies are being more often used in the interventions promoting physical activity [45]. As it has been shown by Kwan et al. [46], a physical activity program with the use of Samsung Health application additionally protects against the occurrence of cognitive function disorders in elderly people. Physical activity-promoting applications may be a valuable support for people aiming at development and maintenance of healthy habits [47]. However, the use of mobile applications may be difficult to use and generate additional stress for the elderly [48]. This problem should be solved by proper instruction on how to use this application during the visit to the general practitioner. Moreover, the use of mobile applications in practice requires further research. It is expected that the IT technologies using the appropriate applications for mobile devices will play a crucial role in healthcare services in the future, enabling the avoidance of direct consultations and providing continuity of health care at the same time [49].

## 5. Limitations

A relatively small number of study subjects may be considered a limitation of this study. In addition, this is the first study that assesses the practical usefulness of the application ECG M-Trace Base II and Samsung Health. In further research, these applications should be compared with other research tools.

Due to the simple operation, the Samsung Health application can be used independently by patients at home. On the other hand, the use of the ECG M-Trace Base II application requires additional training that allows the patient to correctly record the ECG. Additionally, the interpretation of the results requires a medical consultation.

## 6. Conclusions

The ECG M-Trace Base II and Samsung Health are mobile applications that assess cardiological parameters and exercise tolerance parameters in patients after IS with risk factors of further CVD. They can be useful to plan the intensity of exercise in rehabilitation programs. Patients with a history of IS obtain worse results of cardiological parameters and exercise tolerance parameters assessed in SMWT and SCT. These results should be considered in the selection of an appropriate rehabilitation program. Post-stroke patients have risk factors for further CVD events which should be monitored in secondary prophylaxis to reduce the occurrence of recurrent CVD events.

## Figures and Tables

**Table 1:** Group characteristics.

		Post-Stroke Group	Control Group	*p*
Sex *n* (%)	M	18(69.2%)	18(69.2%)	1.0 ^d^
	W	8(30.8%)	8(30.8%)	
Age (years)	Mean ± SD	54.9 ± 7.1	55.6 ± 6.1	0.608 ^c^
	Median	56.5	55	
	Min-Max	42.0–65.0	44.0–63.0	
BMI *	Mean ± SD	29.8 ± 4.9	24.8 ± 3.3	<0.001 ^b^
	Median	29.6	24	
	Min-Max	18.6–37.8	19.6–32.7	
β-blocker taken	Yes	17 (65.4%)	0 (0.0%)	<0.001 ^d^
Hypertension *	Yes	23 (88.5%)	0 (0.0%)	<0.001 ^d^
Diabetes mellitus *	Yes	6 (23.1%)	0 (0.0%)	0.030 ^d^
Dyslipidemia *	Yes	10 (38.5%)	0 (0.0%)	<0.001 ^d^
Atrial fibrillation *	Yes	1 (3.8%)	0 (0.0%)	1.0 ^d^
Depression *	Yes	6 (23.1%)	0 (0.0%)	0.030 ^d^
Insomnia *	Yes	8 (30.8%)	0 (0.0%)	0.002 ^d^
Stenosis of internal carotid artery *	<50%	19 (73.1%)	0 (0.0%)	0.018 ^d^
	50–69%	1 (3.8%)	0 (0.0%)	
	>70%	6 (23.1%)	0 (0.0%)	
Thyroid diseases	Hypothyroidism	1 (3.8%)	0 (0.0%)	0.114 ^d^
	Hyperthyroidism	2 (7.7%)	0 (0.0%)	
Epilepsy	Yes	3 (11.5%)	0 (0.0%)	0.234
Nicotinism *	Yes	6 (23.1%)	0 (0.0%)	0.030 ^d^
Alcoholism *	Yes	1 (3.8%)	0 (0.0%)	1.0 ^d^

BMI–Body mass index ^b^ Welch test, ^c^ Mann–Whitney test, ^d^ chi^2^ test, * risk factors for the reoccurrence of a CVD event.

**Table 2:** Parameters of exercise tolerance.

VARIABLE	SMWT							SCT						
	SG			CG			*p*	SG			CG			*p*
	Mean ± SD	Median	Min-Max	Mean ± SD	Median	Min-Max		Mean ± SD	Median	Min-Max	Mean ± SD	Median	Min-Max	
Fatigue according to Borg	4.9 ± 2.7	5	0.5–9.0	2.2 ± 0.6	2	1.0–3.0	<0.001 ^c^	7.0 ± 2.7	8	2.0–10.0	3.1 ± 0.7	3	2.0–4.0	<0.001 ^c^
Dyspnoea according to Borg	2.1 ± 1.5	2	0.0–6.0	0.0 ± 0.0	0	0.0–0.0	<0.001 ^c^	3.7 ± 2.1	3	0.5–9.0	0.1 ± 0.2	0	0.0–1.0	<0.001 ^c^
Time (min)	5.31 ± 1.03	5.54	2.33–6.60	5.84 ± 0.28	6.01	5.40–6.14	0.044 ^c^	1.78 ± 0.90	1.44	0.49–3.55	0.52 ± 0.15	0.5	0.35–1.01	<0.001 ^c^
Distance (metres)	320 ± 140	370	30–480	550 ± 50	550	450–650	<0.001 ^c^	40 ± 20	50	10–90	50 ± 10	50	40–70	0.179 ^c^
Number of steps	448.2 ± 167.6	512	100.0–631.0	681.3 ± 48.5	670.5	600.0–807.0	<0.001 ^c^	123.9 ± 39.1	126.5	50.0–183.0	85.2 ± 11.6	83.5	67.0–107.0	<0.001 ^b^
Kcal	19.4 ± 6.4	22	7.0–29.0	26.9 ± 2.2	27	23.0–30.0	<0.001 ^c^	8.2 ± 3.0	8	4.0–16.0	4.8 ± 1.0	4.5	4.0–7.0	<0.001 ^c^
Mean velocity (km/h)	3.4 ± 1.2	4	0.3–4.8	5.2 ± 0.5	5.2	4.4–6.4	<0.001 ^c^	2.4 ± 1.0	2.6	0.4–3.9	4.2 ± 0.6	4.3	3.2–5.9	<0.001 ^b^
Max velocity (km/h)	6.1 ± 1.3	6.2	1.3–8.2	7.0 ± 0.7	7	5.6–8.5	0.002 ^c^	5.7 ± 0.9	5.9	3.1–7.1	6.6 ± 1.0	6.5	4.9–8.7	<0.001 ^a^

CG-Control group, Kcal-kilocalories, SCT-Stair climb test, SMWT-Six-minute walk test, SD-Standard deviation, *SG*-Stroke group, ^a^ Student’s *t* test for independent variables ^b^ Welch’s test, ^c^ U Mann–Whitney test.

**Table 3:** Cardiological parameters in the assessment of exercise tolerance.

Variable	Rest			SMWT			SCT			SG				CG			
	SG	CG	*p*	SG	CG	*p*	SG	CG	*p*								
	Mean ± SD	Mean ± SD		Mean ± SD	Mean ± SD		Mean ± SD	Mean ± SD		Rest–SMWT	Rest–SCT	Stairs–SMWT	*p*	Rest–SMWT	Rest–SCT	SCT-SMWT	*p*
SBP	129.9 ± 15.5	128.8 ± 16.0	0.800 ^a^	138.5 ± 11.6	137.5 ± 18.9	0.826 ^b^	145.0 ± 12.0	147.9 ± 18.9	0.200 ^c^	8.6 ± 10.6 *	15.0 ± 9.2 *	6.5 ± 10.2	<0.001	8.7 ± 14.8 *	19.1 ± 14.6 *	10.3 ± 13.9 *	<0.001
DBP	84.6 ± 10.4	81.3 ± 9.9	0.257 ^a^	85.1 ± 6.5	83.5 ± 12.2	0.545 ^b^	83.5 ± 9.0	89.5 ± 13.6	0.068 ^b^	0.5 ± 7.7	−1.1 ± 9.5	−1.7 ± 8.8	0.372	2.8 ± 7.8	8.1 ± 10.4 *	5.3 ± 13.1	0.005
Sat	95.7 ± 1.4	97.0 ± 1.8	0.015 ^c^	95.8 ± 1.2	96.9 ± 1.7	0.023 ^c^	95.3 ± 1.6	96.7 ± 1.6	0.008 ^c^	0.1 ± 1.5	−0.4 ± 2.3	−0.5 ± 1.9	0.982	3.6 ± 19.6	3.3 ± 19.6	−0.2 ± 2.1	0.427
HR	61.7 ± 11.0	66.4 ± 13.0	0.167 ^a^	72.6 ± 10.6	82.7 ± 13.0	0.004 ^a^	80.8 ± 12.5	97.6 ± 16.6	<0.001 ^a^	15.0 ± 16.8 *	19.1 ± 14.1 *	4.2 ± 11.8 *	<0.001	20.8 ± 17.4 *	31.2 ± 20.0 *	10.4 ± 13.9	<0.001
ECG–ischemic features	0 (0%)	0 (0%)	1.0 ^d^	4 (15.4%)	0 (0%)	0.118 ^d^	3 (11.5%)	0 (0%)	0.234 ^d^								

CG-Control group, ECG-electrocardiogram, HR-Heart rate, DBP–Diastolic blood pressure, SBP-Systolic blood pressure, Sat–saturation, SCT-Stair climb test, *SG*-Stroke group, SMWT-Six-minute walk test ^a^ Student’s *t* test for independent variables, ^b^ Welch’s test, ^c^ U Mann–Whitney test, ^d^ chi^2^ test, * Anova Friedman’s test with post-hoc test.

## Data Availability

The data presented in this study are available on request from the first author. The data are not publicly available due to ethical restrictions.
